# TRPV1 Activation Is Associated with Improved Mitochondrial Function and Cardioprotection in Experimental Hypertension

**DOI:** 10.3390/molecules31132212

**Published:** 2026-06-23

**Authors:** Angélica Ruiz-Ramírez, Francisco Correa-Segura, Leonardo Del Valle-Mondragón, Arantxa Marianne Márquez-Ramírez, Israel Pérez-Torres, Oralia Medina Rodríguez, Rodrigo Velázquez-Espejel, Alvaro Vargas-González, Luz Ibarra-Lara, Victor Hugo Oidor-Chan, Julieta Anabell Díaz-Juárez, Raúl Martínez-Memíje, Vicente Castrejón-Téllez, Juan Carlos Torres-Narváez

**Affiliations:** 1 Department of Pharmacology, National Institute of Cardiology Ignacio Chávez, Mexico City 14080, Mexico; angelica.ruiz@cardiologia.org.mx (A.R.-R.); leonardo.delvalle@cardiologia.org.mx (L.D.V.-M.); arantxamarianne@gmail.com (A.M.M.-R.); luz.ibarra@cardiologia.org.mx (L.I.-L.); anabelldij@gmail.com (J.A.D.-J.); 2 Department of Cardiovascular Biomedicine, National Institute of Cardiology Ignacio Chávez, Mexico City 14080, Mexico; francisco.correa@cardiologia.org.mx (F.C.-S.); israel.perez@cardiologia.org.mx (I.P.-T.); 3 Department of Pathology, National Institute of Cardiology Ignacio Chávez, Mexico City 14080, Mexico; oralia_medina@yahoo.com (O.M.R.); rodrigo.velazquez@cardiologia.org.mx (R.V.-E.); 4 Department of Physiology, National Institute of Cardiology Ignacio Chávez, Juan Badiano 1, Sección XVI, Tlalpan, Mexico City 14080, Mexico; alvaro.vargas@cardiologia.org.mx; 5 Department of Biotechnology, Universidad Autónoma Metropolitana-Iztapalapa, Mexico City 09340, Mexico; victorhugooidor@xanum.uam.mx; 6 Department of Electromechanical Instrumentation, National Institute of Cardiology Ignacio Chávez, Mexico City 14080, Mexico; raul.martinez@cardiologia.org.mx

**Keywords:** systemic arterial hypertension, TRPV1, capsaicin, mitochondrial function, oxidative stress, cardioprotection

## Abstract

**Background:** Systemic arterial hypertension (SAH) induced by Nω-nitro-L-arginine methyl ester (L-NAME) is a well-established model characterized by nitric oxide (NO^●^) synthase inhibition and vascular dysfunction. The transient receptor potential vanilloid 1 (TRPV1) regulates Ca^2+^ flux and may contribute to mitochondrial homeostasis. We hypothesized that TRPV1 activation modulates mitochondria function and attenuates cardiac damage during SAH. **Methods:** Hypertension was induced in Wistar rats by administration of L-NAME (200 mg/L) for 40 days. During the last four days, hypertensive animals received capsaicin (5 mg/kg/day), capsazepine (6 mg/kg/day), or their combination. Cardiac function was evaluated in isolated hearts using the Langendorff perfusion system. Myocardial tissue viability was assessed by triphenyltetrazolium chloride (TTC) staining, and mitochondrial function was evaluated by measuring respiratory control and apoptosis-related proteins. **Results:** Capsaicin treatment was associated with significant cardioprotective effects in hypertensive rats. Although the findings are consistent with a role of TRPV1 activation in mediating these effects, the partial protection observed with capsazepine suggests that TRPV1-independent mechanisms may also contribute. **Conclusions:** TRPV1 activation contributes to cardioprotection in SAH, likely through preservation of mitochondrial function and redox balance. However, additional mechanisms beyond TRPV1 modulation may also participate in the observed protective effects. Further studies—including direct assessment of mitochondrial Ca^2+^ flux and the use of more selective or genetic approaches—are currently underway to clarify the underlying mechanisms.

## 1. Introduction

Systemic arterial hypertension (SAH) is a multifactorial disease and a leading cause of cardiovascular morbidity and mortality worldwide [1]. It is associated with the development of severe complications, including target organ damage, heart failure, and myocardial injury. The pathogenesis of SAH involves genetic predisposition, unhealthy dietary habits, and inadequate energy expenditure. Current strategies to mitigate SAH include lifestyle modifications, pharmacological therapy, and the continuous development of novel therapeutic approaches [2,3,4,5].

Hypertension is characterized by structural and biochemical alterations affecting the vascular wall and endothelium, leading to vascular inflammation and dysfunction. These alterations are closely associated with increased production of reactive oxygen species (ROS), including superoxide anion (O_2_^●−^) and peroxynitrite (ONOO^−^) [6,7,8]. Excessive ROS promote endothelial nitric oxide synthase (eNOS) uncoupling through the oxidation of tetrahydrobiopterin (BH4) to dihydrobiopterin (BH2), impairing L-arginine oxidation and reducing nitric oxide (NO^●^) bioavailability, while further increasing O_2_^●−^ production [9,10,11,12].

The transient receptor potential vanilloid type 1 (TRPV1) is a key regulator of intracellular Ca^2+^ homeostasis and NO^●^ signaling in several cell types, mediating Ca^2+^ flux across both plasma and mitochondrial membranes [13,14,15]. TRPV1 is functionally expressed in multiple intracellular organelles, including mitochondria, the Golgi apparatus, and the rough endoplasmic reticulum. Emerging evidence suggests that TRPV1 participates in mitochondrial membrane depolarization [15,16,17], regulation of the mitochondrial permeability transition pore (mPTP), and modulation of pro-apoptotic signaling pathways [17,18,19,20].

TRPV1 activity depends on its cellular localization and exposure to physical and chemical stimuli, which may either activate or inhibit the receptor in neuronal or non-neuronal tissues [15,16,17,18,19,20,21]. Capsaicin (CS), the bioactive compound of chili peppers, is the most widely used exogenous agonist of TRPV1, whereas capsazepine (CZ), a synthetic analog of capsaicin, acts as a selective antagonist and is commonly used to evaluated TRPV1-mediated effects [21,22,23].

Alterations in Ca^2+^ handling are a hallmark of endothelial dysfunction and cardiac injury, leading to decreased NO^●^ bioavailability, ischemia, elevated endothelin-1 and angiotensin-II levels, enhanced ROS generation, and activation of apoptotic pathways [24,25,26,27]. These processes ultimately contribute to acute myocardial infarction (AMI) and cardiac dysfunction. In this context, TRPV1 activation has been associated with cardioprotective effects, partly mediated by the release of neuropeptides such as substance P and the calcitonin gene-related peptide (CGRP), which exert potent vasodilatory actions in concert with NO^●^ signaling and mitochondrial function [13,26,27,28].

Accumulating evidence supports TRPV1 as a potential therapeutic target not only in pain modulation but also in pathological conditions such as hypertension, cancer, and cardiovascular diseases [29,30,31,32]. However, the role of TRPV1 in the regulation of mitochondrial function under hypertensive conditions remains incompletely understood.

In the present study, we investigated whether systemic activation of TRPV1 modulates mitochondrial function in the hearts of hypertensive rats and confers protection against functional and structural cardiac damage. We hypothesized that TRPV1-mediated regulation of mitochondrial Ca^2+^ flux preserves mitochondrial bioenergetics and redox balance, thereby attenuating hypertension-induced myocardial injury.

## 2. Results

Prior to the experimental procedures, mean arterial pressure (MAP) in control (C) rats was 124 ± 3 mmHg, whereas hypertensive (H) rats exhibited a significantly higher MAP of 159 ± 4 mmHg. Treatment with capsaicin partially reduced MAP in the H + CS group (144 ± 6 mmHg), whereas capsazepine treatment resulted in MAP values of 165 ± 5 mmHg.

### 2.1. Cardiac Work

**Figure 1:**
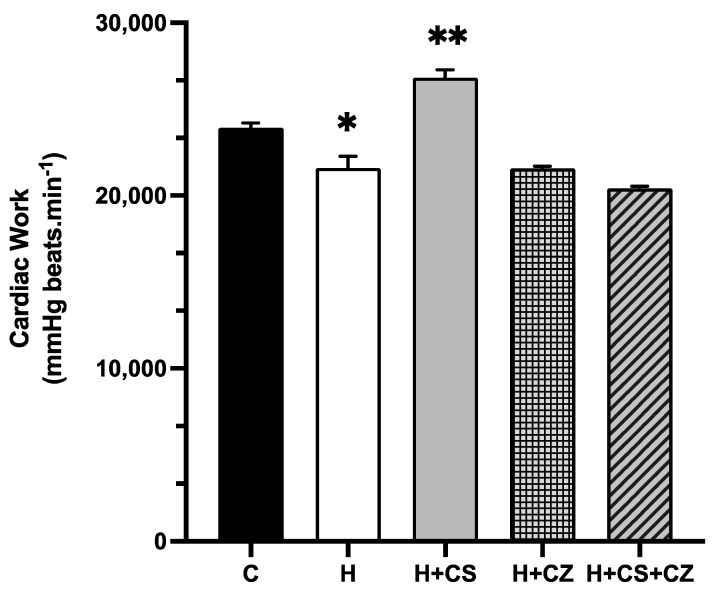
Cardiac work of hypertensive rats. Statistical analysis was performed using one-way ANOVA followed by Tukey’s post hoc (*p* < 0.05). Values are expressed as mean ± SE (*n* = 8 per group). *, C vs. H; **, H vs. H + CS.

Figure 1 shows that cardiac work (CW) was significantly reduced in isolated hearts from hypertensive rats (21,598 ± 1179 mmHg·beats·min^−1^) compared with control hearts (23,920 ± 507 mmHg·beats·min^−1^). TRPV1 activation by capsaicin significantly improved CW in hypertensive rats (H + CS), increasing values to 26,823 ± 792 mmHg·beats·min^−1^ relative to the H group.

In contrast, blockade of TRPV1 with capsazepine did not improve cardiac work. Hearts from the H + CZ and H + CS + CZ groups exhibited reduced CW values of 21,569 ± 540 and 20,418 ± 637 mmHg·beats·min^−1^, respectively, compared with the H + CS group.

### 2.2. Coronary Vascular Resistance

**Figure 2:**
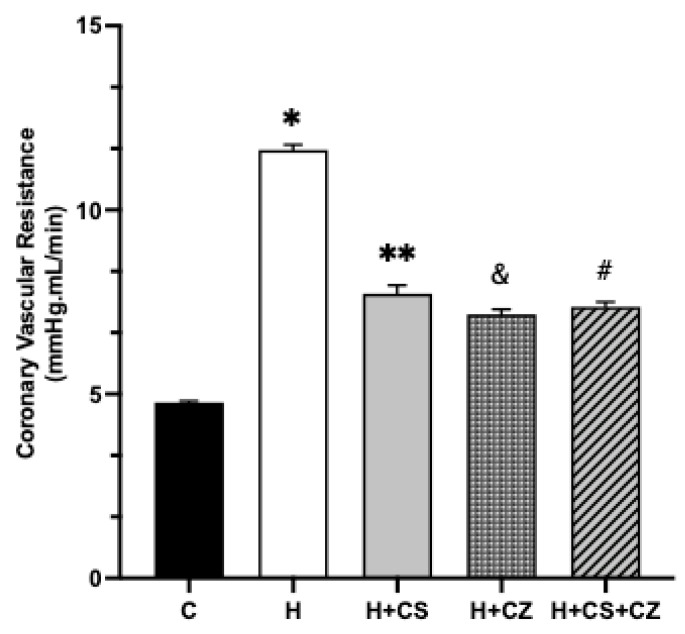
Effect of hypertension on coronary vascular resistance. Statistical analysis was performed using one-way ANOVA followed by Tukey’s post hoc test (*p* < 0.05). Values are expressed as mean ± SE (*n* = 8 per group). * C vs. H; ** H vs. H + CS; &, H vs. H + CZ or #; H + CS + CZ.

Figure 2 shows that the coronary vascular resistance (CVR) was markedly increased in hypertensive rats compared with controls, rising from 4.77 ± 0.06 to 11.63 ± 0.25 mmHg·mL^−1^·min^−1^. TRPV1 activation significantly decreased CVR in the H + CS group (7.73 ± 0.39 mmHg·mL^−1^·min^−1^) relative to untreated hypertensive rats.

In hypertensive rats treated with capsazepine or combined CS + CZ treatment, CVR values were 7.17 ± 0.23 and 7.37 ± 0.22 mmHg·mL^−1^·min^−1^, respectively, remaining comparable to those observed in the H + CS group, showing significantly differences like CS group I hypertensive rats.

### 2.3. TTC-Negative Myocardial Area

**Figure 3:**
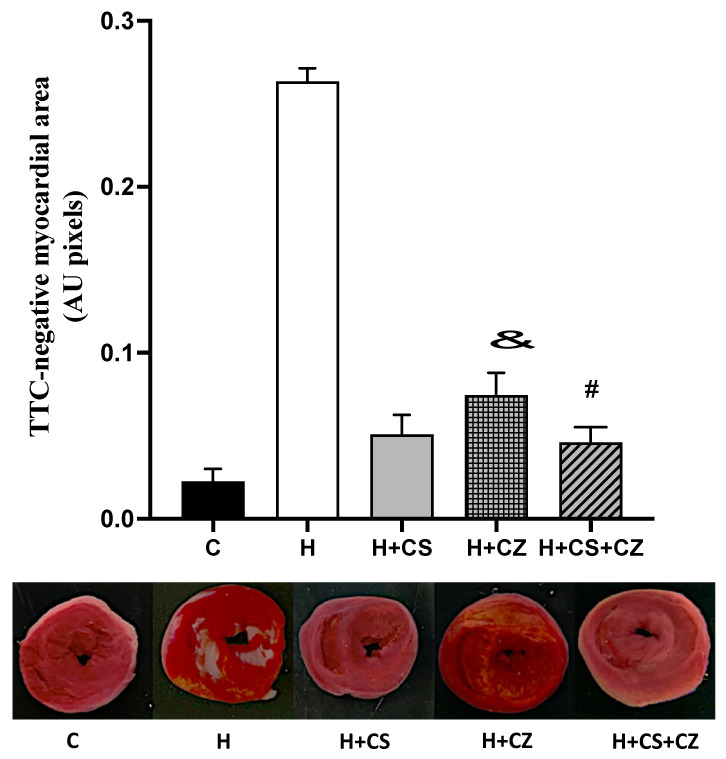
Effect of capsaicin on TTC-negative myocardial area in hypertensive rat hearts. Hearts were stained with triphenyltetrazolium chloride. TTC-negative myocardial area was expressed as the ratio of damage area to area at risk, as described in Materials and Methods. Results are expressed as mean ± SE. (*n* = 3 per group). Statistical analysis was performed using one-way ANOVA followed by Tukey’s post hoc test (*p* < 0.05), &, H + CZ or # H + CS + CZ.

To further evaluate cardiac damage in hypertensive animals and the effect of CS, CZ and CS + CZ treatments, TTC-negative myocardial area was determined in all experimental groups. Hypertensive rats exhibited a significantly increased in TTC-negative myocardial area (0.2635 ± 0.007) compared with group controls (0.0225 ± 0.007). Capsaicin treatment significantly reduced TTC-negative myocardial area (0.050 ± 0.01). Similarly, CZ (0.075 ± 0.01) and CS + CZ (0.046 ± 0.009) treatments also significantly decreased TTC-negative myocardial area, as shown in Figure 3.

### 2.4. Histological Analysis of Myocardial Damage

**Figure 4:**
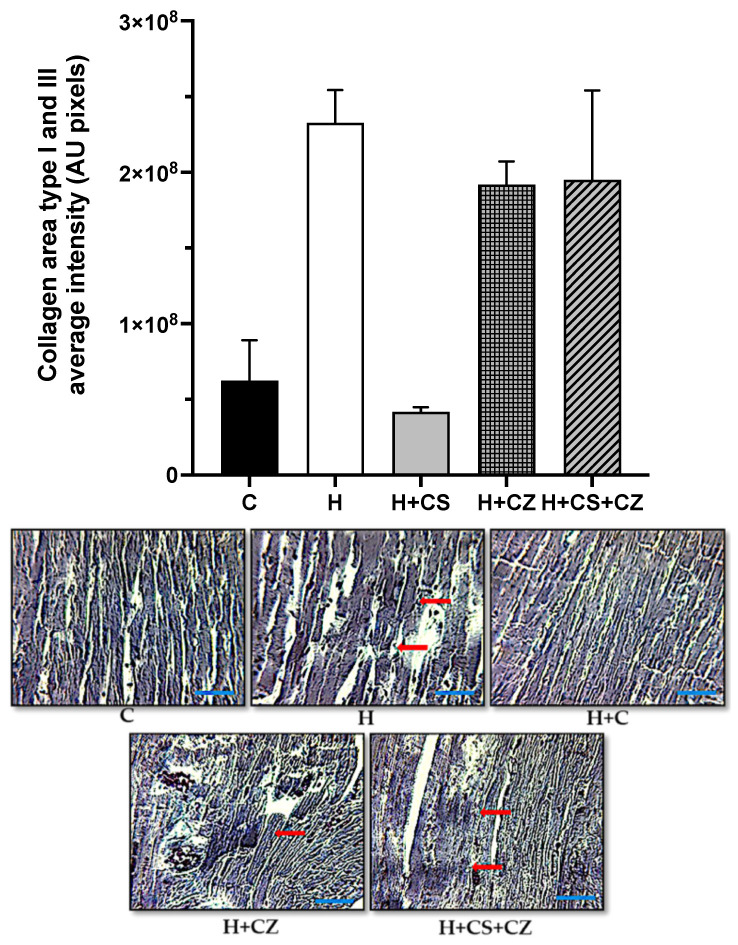
Representative photomicrographs (20×) and densitometric analysis of collagen types I and III deposition in myocardial tissue. Red arrows indicate collagen deposition. Values are expressed as mean ± SE (arbitrary units, *n* = 6). Statistical analysis was performed using one-way ANOVA followed by Tukey’s post hoc test. Sections were stained with Sirius Red. Scale bar = 50 μm.

Representative histological sections are shown in Figure 4. Control hearts displayed well-organized myocardial architecture, characterized by compactly arranged cardiomyocytes, preserved intercalated discs, and minimal interstitial fibrosis. In contrast, hypertensive hearts exhibited tissue damage, increased fibrosis, and structural disorganization.

Capsaicin treatment markedly attenuated these alterations, resulting in myocardial morphology comparable to that of control hearts. In contrast, hearts from the H + CZ group showed moderate necrosis, increased fibroblast and macrophage infiltration, and areas of hypertrophy and myocardial injury. The H + CS + CZ group exhibited limited fibroblast and lymphocyte proliferation, with fewer structural alterations compared with the H group.

Quantitative densitometric analysis revealed a significant increase in collagen types I and III deposition in the H, H + CZ, and H + CS + CZ groups. Notably, collagen accumulation between myocardial fibers was markedly attenuated in the H + CS group, indicating a protective effect of TRPV1 activation (Figure 4).

### 2.5. Cardiac Mitochondrial Function

Mitochondria isolated from hypertensive hearts exhibited a significant reduction in the respiratory control ratio (RC) compared with controls (Table 1). Capsaicin treatment significantly restored RC in the H + CS group, whereas capsazepine treatment did not improve mitochondrial coupling. Interestingly, combined CS + CZ treatment restored RC values to control levels.

Consistent with these findings, ADP/O ratios followed a similar pattern. State III respiration rates were highest in mitochondria from the H + CS group, whereas mitochondria from the H, H + CZ, and H + CS + CZ groups showed oxygen consumption rates comparable to controls. Under State IV conditions, oxygen uptake was significantly increased in hypertensive mitochondria, indicating mitochondrial uncoupling. In contrast, combined CS + CZ treatment significantly reduced oxygen consumption relative to the H group.

### 2.6. Apoptotic Protein Expression

**Figure 5:**
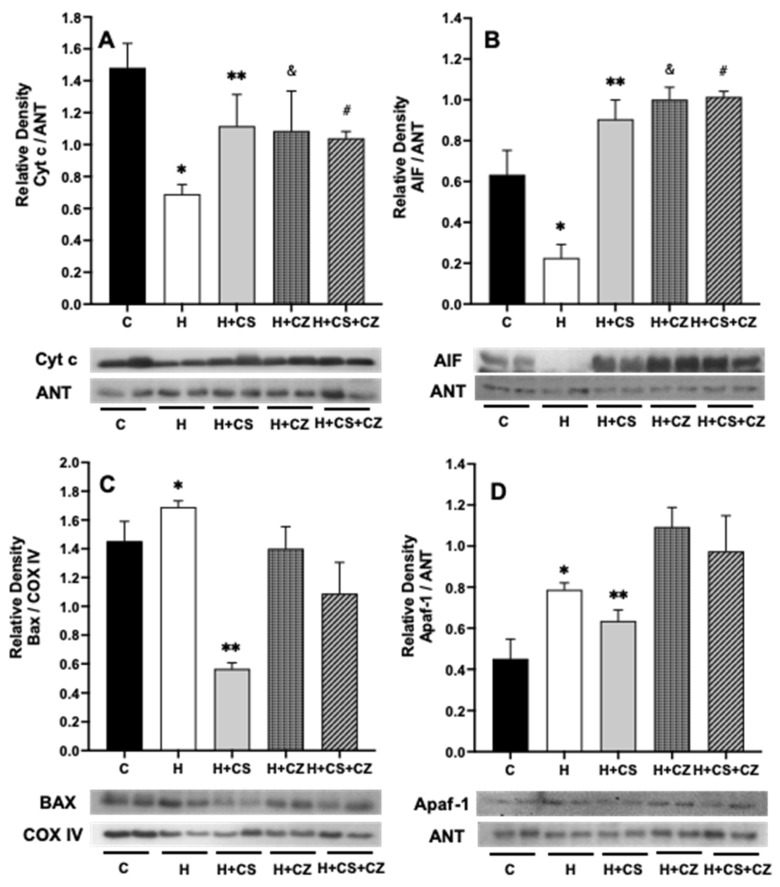
Western blots analysis of cytochrome *c* (**A**), AIF (**B**), Bax (**C**), and Apaf-1 (**D**) in isolated cardiac mitochondria. Statistical analysis was performed using one-way ANOVA followed by Tukey’s post hoc test (*p* < 0.05). Values are expressed as mean ± SE (*n* = 4) (*p* ≤ 0.01) * C vs. H; (*p* < 0.05) ** H vs. CS, & H vs. H + CZ; # CS + CZ groups.

Western blot analysis showed a significant reduction in mitochondrial cytochrome *c* levels in the H group compared with controls, suggesting its release into the cytosol (Supplementary Materials). In contrast, cytochrome *c* levels were preserved in mitochondria from the H + CS, H + CZ, and H + CS + CZ groups (Figure 5A).

Mitochondrial apoptosis-inducing factor (AIF) content was significantly reduced in hypertensive rats compared with controls, indicating its translocation to the cytosol. Treatment with CS, CZ, and CS + CZ preserved mitochondrial AIF levels (Figure 5B).

Figure 5C shows a significant increase in Bax insertion into mitochondria in hypertensive rats compared with controls, whereas capsaicin treatment reduced BAX mitochondrial localization, suggesting inhibition of apoptotic signaling.

Apaf1 mitochondrial levels were significantly decreased in the H + CS group compared with all other groups, indicating attenuation of intrinsic apoptotic signaling following TRPV1 activation (Figure 5D).

### 2.7. Antioxidant Enzyme Activity

**Figure 6:**
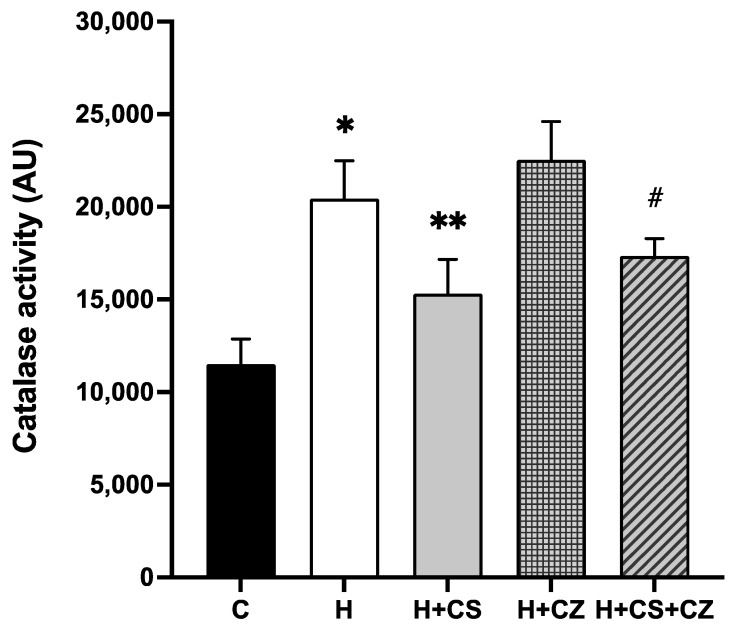
Catalase activity analyzed by native gel electrophoresis and corresponding densitometric quantification. Intense bar. Statistical analysis was performed using one-way ANOVA followed by Tukey’s test. Values are expressed as mean ± SE (*n* = 4). * *p* ≤ 0.001, C vs. H; ** *p* ≤ 0.01, H vs. CS; # *p* ≤ 0.05 H vs. CS + CZ groups. St = Standard activity.

Catalase activity was significantly increased in the hypertensive group compared with controls. Treatment with Capsaicin significantly reduced catalase activity, whereas CZ had no significant effect. The increase in catalase activity observed in hypertensive animals may reflect an adaptive antioxidant response to elevated oxidative stress (Figure 6).

## 3. Discussion

Activation of the TRPV1 receptor by capsaicin exerts a significant cardioprotective effect in L-NAME-induced hypertension. The protective response was observed at functional, structural, and molecular levels, supporting an important role of TRPV1 in preserving cardiac homeostasis under hypertensive conditions. Hypertensive animals exhibited impaired cardiac work, increased coronary vascular resistance, myocardial injury, collagen deposition, mitochondrial dysfunction, and activation of apoptotic pathways. In contrast, capsaicin treatment attenuated these alterations, suggesting that TRPV1 activation contributes to the maintenance of cardiac function during chronic nitric oxide deficiency.

Due to the multifactorial nature of systemic arterial hypertension (SAH), several animal models have been developed to reproduce its complications and to explore potential therapeutic strategies. Hypertension induced by high doses of L-NAME is a well-established experimental model in which the activity of the three nitric oxide synthase (NOS) isoforms—neuronal, inducible, and endothelial—is nonspecifically inhibited, resulting in reduced nitric oxide (NO^●^) bioavailability [6,8,12,25].

To evaluate the role of TRPV1 in myocardial protection under hypertensive conditions, capsaicin (CS), a potent exogenous TRPV1 agonist, was used. We investigated whether TRPV1 activation promotes cardiac recovery from hypertension-induced damage through regulation of mitochondrial metabolism. As expected, TRPV1 activation by CS exerted a protective effect on cardiac work, preventing the functional deterioration observed in hypertensive rats. In contrast, capsazepine (CZ) alone reduced cardiac work by approximately 20% to control animals and abolished the beneficial effects of CS when both compounds were administered together.

Accumulating evidence indicates that TRPV1 channels participate in multiple cardioprotective mechanisms, including the regulation of oxidative stress, inflammation, mitochondrial function, calcium homeostasis, and cell survival pathways. Recent reviews have highlighted the potential therapeutic relevance of TRPV1 activation in several cardiac pathologies, including hypertension, myocardial ischemia–reperfusion injury, cardiac hypertrophy, and heart failure [33].

Hypertensive rats exhibited a significant increase in coronary vascular resistance (CVR) compared with controls. TRPV1 activations by CS markedly reduced this parameter, suggesting a protective vascular effect. Interestingly, CZ treatment alone also reduced coronary vascular resistance, and co-administration of CS and CZ did not abolish this effect. Therefore, the improvement in CVR observed in the present study cannot be attributed exclusively to TRPV1 activation. These findings suggest that TRPV1-independent mechanisms may also contribute to vascular protection. Capsazepine has been reported to exert biological effects beyond TRPV1 antagonism, including antioxidant and anti-inflammatory actions, which may improve vascular function under conditions of oxidative stress. Because L-NAME-induced hypertension is characterized by endothelial dysfunction and excessive reactive oxygen species production, it is possible that the reduction in CVR observed in the CZ-treated groups resulted, at least in part, from these additional pharmacological properties. Consequently, although TRPV1 activation may participate in the vascular effects observed with capsaicin, the present results do not allow a definitive distinction between TRPV1-dependent and TRPV1-independent mechanisms. Future studies employing genetic models or more selective TRPV1 inhibitors will be necessary to clarify the specific role of TRPV1 in the regulation of coronary vascular resistance during hypertension [34,35,36].

Although the present study supports a cardioprotective role of TRPV1 activation, previous studies have reported that selective sensory desensitization induced by capsaicin may adversely affect cardiac function. For example, desensitization of capsaicin-sensitive sensory nerves has been associated with increased left ventricular end-diastolic pressure and impaired myocardial relaxation in isolated working heart preparations. These findings highlight the complex role of TRPV1-related pathways in cardiovascular regulation and suggest that the physiological consequences of capsaicin exposure may depend on dose, duration of treatment, and the balance between receptor activation and sensory nerve desensitization [37].

Histological analysis corroborated the functional findings. Hypertensive rats treated with CS exhibited preserved myocardial architecture comparable to that of control animals, whereas untreated hypertensive hearts showed marked structural damage. The cardioprotective effect of CS is likely mediated, at least in part, by TRPV1 activation, which reduces oxidative stress, regulates Ca^2+^ flux, and restores NO^●^ signaling, as previously reported [25]. Furthermore, CS treatment markedly reduced collagen deposition in ventricular tissue, a key factor in maintaining myocardial mechanical properties. An additional consideration is the relatively short duration of capsaicin and capsazepine treatment compared with the 40-day hypertensive period. Therefore, the histological improvements observed in the treated groups should not be interpreted as complete reversal of established cardiac remodeling. Instead, these findings likely reflect attenuation of ongoing myocardial injury and preservation of tissue viability through mechanisms involving improved coronary perfusion, reduced oxidative stress, preservation of mitochondrial function, and suppression of apoptotic signaling. Since myocardial injury is a dynamic process, modulation of these pathways may produce detectable functional and structural benefits even after a relatively short intervention period. Nevertheless, longer treatment protocols will be necessary to determine whether sustained TRPV1 modulation can induce more extensive regression of hypertension-associated cardiac remodeling.

TTC-negative myocardial area, a well-established marker of myocardial injury, further supported these findings. TRPV1 activation and the antioxidant properties of capsaicin significantly reduced TTC-negative myocardial area, indicating protection against oxidative stress and NO^●^ depletion associated with hypertension. Although CZ also reduced TTC-negative myocardial area, this unexpected effect may be explained by its antioxidant activity and other reported pharmacological properties [22,37,38]. A limitation of the present study is that TTC staining was used to evaluate myocardial tissue viability in hearts obtained from hypertensive animals without the induction of an experimental ischemic event. Therefore, TTC-negative regions should not be interpreted as classical myocardial infarction or infarct size, as commonly defined in ischemia–reperfusion or coronary occlusion models. Instead, these areas likely reflect myocardial tissue injury and loss of cellular viability associated with chronic hypertension-induced cardiac damage. Accordingly, TTC findings were interpreted as an index of myocardial injury and were considered together with the functional, histological, and mitochondrial alterations observed in this study.

Mitochondrial metabolism is the primary source of energy in cardiomyocytes, and mitochondrial dysfunction—characterized by impaired electron transport chain efficiency and reduced ATP production—is a hallmark of chronic diseases, including hypertension [39,40,41]. In addition, dysfunctional mitochondria are a major source of reactive oxygen species (ROS), further exacerbating oxidative stress [41,42]. Our results demonstrated a significant reduction in respiratory control (RC) and ATP synthesis in hypertensive rats, both of which were restored by CS treatment. These findings suggest that TRPV1 activation improves mitochondrial efficiency and preserves bioenergetic function under hypertensive conditions. This interpretation is consistent with previous reports showing that TRPV1 regulates mitochondrial membrane potential via calcineurin-dependent signaling in cardiomyocytes [43,44].

Mitochondrial dysfunction and oxidative stress are closely associated with the release of cytochrome *c* (Cyt *c*) into the cytosol, triggering apoptosis [45,46]. The reduced mitochondrial Cyt *c* content observed in hypertensive animals suggests its translocation into the cytosol, consistent with enhanced apoptotic signaling, as reported in other hypertensive models [47]. Similarly, mitochondrial levels of apoptosis-inducing factor (AIF) were significantly decreased in hypertensive rats, indicating its release into the cytosol; this effect was prevented by CS treatment.

Cytochrome *c* is a hemoprotein that functions as an electron carrier between complexes III and IV of the mitochondrial respiratory chain. Under conditions of increased ROS, its interaction with cardiolipin is disrupted, allowing its release from mitochondria and activation of apoptotic pathways [48]. AIF, a flavoprotein associated with cytochrome *c* oxidase, also plays a role in redox regulation, and its release is directly linked to cell death processed [49]. The concomitant decrease in Cyt *c* and AIF in mitochondria, together with impaired respiratory control, supports the occurrence of apoptosis in hypertensive rats. Notably, TRPV1 activation by CS reversed these alterations, thereby preserving mitochondrial integrity and preventing cell death.

Apaf-1 is a cytosolic apoptotic protein that, upon activation, translocates to mitochondria to associate with cytochrome *c* and caspase-9, forming the apoptosome. Our results showed a significant increase in mitochondrial Apaf-1 levels in hypertensive rats, whereas CS treatment prevented its accumulation, indicating suppression of intrinsic apoptotic signaling.

The release of cytochrome *c* and AIF may occur through mitochondrial permeability transition pore (mPTP) opening or via Bax-mediated pore formation in the outer mitochondrial membrane. In agreement with this, our results showed a significant increase in BAX insertion into mitochondrial membranes in hypertensive rats, whereas TRPV1 activation reduced its localization, further supporting a protective anti-apoptotic effect of CS. The results are consistent with the possibility that TRPV1 activation contributes to preservation of mitochondrial function and attenuation of apoptotic signaling. Similar anti-apoptotic effects of TRPV1 activation have been reported in ischemia–reperfusion models via AKT/ERK signaling pathways [50].

The increased oxygen consumption observed in H + CS during State III respiration may reflect mild mitochondrial uncoupling mediated by uncoupling proteins (UCPs), which are upregulated under oxidative stress conditions such as hypertension [44,51]. UCP2 and UCP3, expressed in cardiac tissue, regulate superoxide production by promoting proton leak and reducing electron leakage toward ROS formation. TRPV1 and thermogenic UCPs are also involved in Ca^2+^ signaling, NO synthesis, and ROS modulation. Although their interaction has been primarily studied in obesity models [52], increasing evidence suggests that UCP2 and UCP3 contribute to mitochondrial ROS regulation [14].

Interestingly, although CZ tended to reduce respiratory control, mitochondrial function was restored to near-control levels in the CS + CZ group. Interestingly, the H + CS + CZ group even showed a non-significant trend toward higher value, although this difference did not reach statistical significance due to the variability within the group. While the precise mechanism underlying this finding remains unclear, several explanations may be considered. Both capsaicin and capsazepine contain phenolic moieties that have been associated with antioxidant activity and free-radical scavenging properties. Therefore, the combined administration of these compounds may provide additive protection against oxidative damage to mitochondrial membranes and respiratory chain components, thereby preserving mitochondrial coupling efficiency. Additionally, it cannot be excluded that the dose of capsazepine employed in this study resulted in only partial TRPV1 inhibition, allowing residual receptor activity in the presence of capsaicin. Given the complexity of TRPV1 signaling and the reported TRPV1-independent actions of capsazepine, further studies using more selective pharmacological tools or genetic approaches will be required to clarify the mechanisms responsible for the enhanced mitochondrial coupling observed in this group [21,38]. Alternatively, the concentration of CZ used may have resulted in only partially inhibited TRPV1 activity.

Hypertension-induced oxidative stress also alters antioxidant enzyme activity. Catalase activity was significantly increased in hypertensive rats, which may represent a compensatory adaptive response to the elevated production of reactive oxygen species associated with chronic nitric oxide deficiency and mitochondrial dysfunction. Although antioxidant enzymes are frequently reported to be decreased under conditions of prolonged oxidative stress due to oxidative inactivation, increased catalase activity has also been described as an early or adaptive response aimed at limiting hydrogen peroxide accumulation. In this context, the reduction in catalase activity observed following capsaicin treatment should not be interpreted as a loss of antioxidant defense, but rather as a consequence of decreased oxidative burden and a reduced requirement for compensatory upregulation of antioxidant enzymes. These findings are consistent with the improved mitochondrial function and respiratory control observed in capsaicin-treated animals. In contrast, catalase activity remained elevated in the H + CZ and H + CS + CZ groups. Similar compensatory increases in catalase activity have been reported in renal hypertension models [53].

A notable finding of this study is the partial protective effect observed with capsazepine (CZ) in certain parameters, such as coronary vascular resistance and myocardial injury, despite its role as a TRPV1 antagonist. This unexpected response suggests that CZ may exert TRPV1-independent effects, potentially related to its intrinsic antioxidant properties, as previously reported. Given its phenolic structure, CZ may act as a free radical scavenger, thereby attenuating oxidative stress and contributing to the observed cardioprotective effects [54,55]. However, this dual behavior also represents a limitation in the interpretation of the results, as it complicates the clear attribution of all observed effects exclusively to TRPV1 modulation.

In addition, although our findings support a role for TRPV1 in the regulation of mitochondrial function, the precise mechanisms involved remain to be fully elucidated. In particular, the lack of direct measurements of mitochondrial Ca^2+^ flux and the absence of genetic or highly selective pharmacological approaches (e.g., TRPV1 knockout models or more specific inhibitors) limit the ability to establish a definitive causal relationship between TRPV1 activation and mitochondrial protection [56,57]. Therefore, future studies should address these aspects to better define the molecular pathways underlying TRPV1-mediated cardioprotection under hypertensive conditions.

In conclusion, our findings suggest that TRPV1 contributes to the regulation of mitochondrial function and myocardial protection under hypertensive conditions. An additional consideration is the relatively short duration and cumulative dose of capsaicin administration used in the present study. The treatment protocol was designed to evaluate the acute effects of TRPV1 activation once hypertension had already been established. Although no overt adverse effects were observed, capsaicin may induce systemic physiological responses, including thermoregulatory, sensory, and autonomic effects, which could potentially contribute to the observed cardioprotective outcomes. Therefore, the possibility that some of the beneficial effects are mediated, at least in part, through systemic mechanisms cannot be completely excluded. Further studies comparing different treatment durations and employing more selective approaches will be necessary to distinguish direct cardiac effects from systemic TRPV1-mediated responses. Although the precise mechanisms remain to be fully elucidated, future studies should explore the potential interaction between TRPV1, UCPs, and mitochondrial regulators such as Sirtuin-3 [58].

## 4. Materials and Methods

### 4.1. Reagents

All reagents used in this study were purchased from Sigma-Aldrich (St. Louis, MO, USA) unless otherwise specified. Capsaicin (CS; 8-methyl-N-vanillyl-6-nonenamide), capsazepine (CZ; *N*-(2-(4-chlorophenyl)ethyl)-1,3,4,5-tetrahydro-7,8-dihydroxy-2H-2-benzazepine-2-carbothioamide), and Nω-nitro-L-arginine methyl ester (L-NAME) were of analytical grade.

### 4.2. Animals

Male Wistar rats (300–350 g) were obtained from the animal facility of the National Institute of Cardiology Ignacio Chávez (Mexico City, Mexico). All experimental procedures were approved by the Institutional Ethics Committee for the Use and Care of Laboratory Animals and conducted in accordance with the Mexican Official Standard for Animal Care (NOM-062-ZOO-1999).

Animals were housed under controlled environmental conditions (12 h light/dark cycle, 25 ± 3 °C, 50 ± 10% humidity) and had free access to water and a standard laboratory diet (LabDiet 5026, PMI Nutrition International, Richmond, IN, USA).

### 4.3. Experimental Design and Treatments

Animals were randomly divided into five experimental groups (*n* = 8 per group):

(1) Control (C);

(2) Hypertensive (H);

(3) Hypertensive + Capsaicin (H + CS);

(4) Hypertensive + Capsazepine (H + CZ);

(5) Hypertensive + Capsaicin + Capsazepine (H + CS + CZ).

Systemic arterial hypertension was induced in groups H to H + CS + CZ by administration of L-NAME (200 mg/L) in drinking water for 40 consecutive days. Mean arterial pressure was measured at baseline and at the end of the experimental period using a non-invasive tail-cuff method.

**Figure 7:**
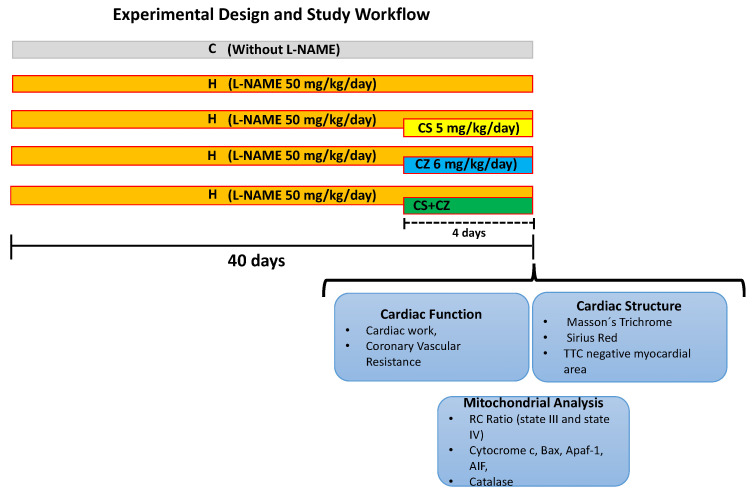
Timeline of experimental protocol. Hypertension was induced by administration of L-NAME for 40 days. Capsaicin (CS), capsazepine (CZ), or the combination of both compounds (CS + CZ) were administered during the final 5 days of the protocol (days 36–40). At day 40, hearts were isolated and processed for functional, histological, biochemical, and mitochondrial analyses.

From day 36 to 40, animals received daily subcutaneous injections as follows: CS (5 mg/kg/day), CZ (6 mg/kg/day), or a combination of CZ followed by CS one hour later. The cumulative doses were 20 mg/kg for CS and 24 mg/kg for CZ. L-NAME treatment was maintained throughout the experimental protocol (Figure 7). Animals were euthanized on day 40. The duration of capsaicin treatment was selected to evaluate the acute effects of TRPV1 activation after the establishment of hypertension rather than to prevent disease development. This dosing regimen was based on our previous studies using the same experimental model, in which significant cardiovascular and endothelial effects were observed without evidence of overt toxicity [25,59,60,61].

Capsaicin and capsazepine were dissolved in an ethanol:water solution (2:1, *v*/*v*).

### 4.4. Isolated and Perfused Heart (Langendorff Preparation)

Rats were anesthetized with sodium pentobarbital (60 mg/kg, i.p.) and subjected to tracheotomy for mechanical ventilation. After thoracotomy, heart was rapidly excised and immersed in ice-cold Krebs–Henseleit (K–H) buffer containing (mM): NaCl 118, KCl 4.7, CaCl_2_ 2.0, MgSO_4_ 1.2, KH_2_PO_4_ 1.2, EDTA 0.5, NaHCO_3_ 25, and glucose 11 (pH 7.4), equilibrated with 95% O_2_ and 5% CO_2_.

The aorta was cannulated, and hearts were perfused retrogradely at 37 °C using a Langendorff apparatus. Coronary flow was maintained at 13 mL/min using a peristaltic pump (SAD22, Grass Instruments Co., Quincy, MA, USA). After a 30 min stabilization period, cardiac parameters were recorded.

Heart rate was electrically paced at 312–324 beats/min using a Grass stimulator (U7). Left intraventricular pressure (LIVP) was measured using a latex balloon inserted into the left ventricle and connected to a pressure transducer. Perfusion pressure (PP) was continuously monitored, and only hearts with baseline PP values between 55 and 70 mmHg were included.

Cardiac work (CW) was calculated as HR × LIVP, and coronary vascular resistance (CVR) as PP/flow [62].

### 4.5. Histological Analysis

Following perfusion, hearts were fixed in 10% formaldehyde and processed for histological analysis. Sections were stained with Masson’s trichrome to assess myocardial structure and fibrosis, and Sirius Red to evaluate collagen deposition.

Images were acquired using a Carl Zeiss Axio Imager Z2 microscope equipped with EC Plan-Neofluar objectives (10× and 20×). Quantitative densitometric analysis was performed using SigmaScan Pro 5 software, and results were expressed as arbitrary pixel density units.

### 4.6. TTC-Negative Myocardial Area

Triphenyltetrazolium chloride (TTC) staining was used to assess myocardial tissue viability. TTC-negative regions were quantified using ImageJ software 1.54g and expressed in pixels as the quotient of the total area at risk (tissue slice)/damaged area (white area). TTC-negative myocardial area (*n* = 3 per group). Hearts were frozen at −20 °C for 24 h and then cut into ~3-mm transverse slices. Slices were incubated in phosphate buffer (88 mM Na_2_HPO_4_ and 1.8 mM NaH_2_PO_4_ at pH 7.4) containing 1% TTC for 10 min at 37 °C, followed by fixation in formalin for 5 min.

Slices were scanned using a Hewlett-Packard Scanjet 3800 scanner (Hewlett-Packard, Palo Alto, CA, USA). Myocardial injury and risk areas were quantified using ImageJ software (National Institutes of Health, USA). Areas were expressed as pixel counts, and myocardial injury was calculated as the ratio of TTC-negative area to total myocardial area analyzed [63].

### 4.7. Isolation of Cardiac Mitochondria

Cardiac mitochondria were isolated by differential centrifugation from six hearts per group. Tissue was homogenized in ice-cold isolation buffer containing 250 mM sucrose, 10 mM HEPES, and 1 mM EGTA (pH 7.3). Homogenates were incubated with proteinase type XXIV (1 mg/mL) for 10 min and centrifuged at 600× *g* for 10 min to remove debris. The supernatant was centrifuged at 8000× *g* for 10 min to obtain the mitochondrial pellet.

Mitochondria were washed and resuspended in isolation buffer containing 0.1% bovine serum albumin (BSA), followed by a final wash without BSA. Isolated mitochondria were kept at 4 °C and used immediately for functional assays [64].

### 4.8. Assessment of Mitochondrial Function

#### 4.8.1. Mitochondrial Respiration

Oxygen consumption was measured using a Clark-type oxygen electrode at 30 °C. Mitochondria (1 mg protein/mL) were incubated in respiration buffer containing 125 mM KCl, 10 mM HEPES (pH 7.4), 10 mM EGTA, and 2 mM K_2_HPO_4_. Glutamate/malate (5/3 mM) were used as substrates. State 3 respiration was initiated by the addition of ADP (250 μM), and State 4 respiration was recorded after ADP depletion [65].

#### 4.8.2. Apoptotic Protein Analysis

Mitochondrial proteins (80 μg) were analyzed by Western blotting to assess cytochrome *c*, Bax, Apaf-1, and AIF expression. Proteins were separated by 12% SDS–PAGE and transferred to PVDF membranes. Membranes were blocked with 1% casein and incubated with primary antibodies against the target proteins, with adenine nucleotide translocator (ANT) or VDAC used as loading controls.

Immunodetection was performed using HRP-conjugated secondary antibodies and enhanced chemiluminescence (SuperSignal™, Thermo Fisher Scientific Inc.168 Third Avenue, Waltham, MA 02451, USA). Band intensities were quantified using ImageJ software 1.54g.

### 4.9. Catalase Activity

Catalase activity was assessed by native polyacrylamide gel electrophoresis. Mitochondrial proteins (60 μg) were separated on 8% native gels at 100 V for 4 h at 4 °C. Gels were incubated with 20 mM H_2_O_2_ followed by staining with potassium ferricyanide and ferric chloride (1% *v*/*v*). Catalase activity appeared as achromatic bands on a blue background [66].

### 4.10. Statistical Analysis

Data are presented as mean ± standard error (SE). Statistical analyses were performed using one-way ANOVA followed by Tukey’s post hoc test, using SigmaPlot version 11. Differences were considered statistically significant at *p* < 0.05.

## 5. Conclusions

In conclusion, TRPV1 may represent a potential therapeutic target for preventing mitochondrial dysfunction and cardiac damage associated with systemic arterial hypertension. Furthermore, the unexpectedly elevated respiratory control ratio observed in the H + CS + CZ group suggests that mechanisms independent of TRPV1 inhibition, including potential antioxidant actions of capsazepine, may contribute to the preservation of mitochondrial function.

Additionally, TTC staining was used to assess myocardial tissue viability in the absence of an experimentally induced ischemic event. Therefore, the TTC-negative areas identified in this study should be interpreted as indicators of myocardial injury rather than classical infarct size.

However, further studies are required to elucidate the underlying molecular mechanisms, including the interaction of TRPV1 with mitochondrial regulatory proteins such as uncoupling proteins and Sirtuin-3, as well as to evaluate the translational relevance of these findings.

## 6. Limitations

It is important to acknowledge certain limitations of the present study. Although capsazepine (CZ) was used as a pharmacological antagonist of TRPV1, its partial protective effects observed in some parameters suggest the possibility of TRPV1-independent actions, potentially related to its intrinsic antioxidant properties. This aspect may limit the precise attribution of the observed effects exclusively to TRPV1 modulation.

Additionally, the absence of direct measurements of mitochondrial Ca^2+^ flux and the lack of genetic or highly selective approaches to specifically inhibit TRPV1 restrict the ability to establish a definitive causal relationship between TRPV1 activation and mitochondrial protection.

These aspects are currently being addressed as part of the ongoing continuation of this work, with the aim of further elucidating the molecular mechanisms involved and strengthening the translational relevance of these findings.

## Figures and Tables

**Table 1:** Mitochondria oxygen uptake.

	C	H	H + CS	H + CZ	H + CS + CZ
State III	277.0 ± 4.9	296.6 ± 47.7	522.6 ± 35.8 **	355.0 ± 12.9	199.4 ± 29.9
State IV	55.9 ± 1.4	137.3 ± 26.6 *	117.6 ± 16.4	111.9 ± 9.3	37.5 ± 10.3 **^#^**
RC	4.96 ± 0.1	2.24 ± 0.21 *	3.90 ± 0.21 **	3.22 ± 0.2 ^&^	5.8 ± 0.82 **^#^**
ADP/O	2.1 ± 0.01	1.5 ± 0.2 *	2.2 ± 0.1 **	1.9 ± 0.3 ^&^	2.5 ± 0.1 **^#^**

Values are expressed as mean ± SE (*n* = 4). State III respiration correspond to ADP-stimulated respiration, whereas State IV represents oxygen consumption in the absence of ADP. Respiratory control (RC) is defined as the State III/State IV ratio. Oxygen consumption is expressed as nmol/min/mg protein. Experiments were performed in the presence of 5 mM glutamate, 3 mM malate, and 250 mM ADP. The ADP/O ratio represents ATP synthesis relative to oxygen consumption. Statistical analysis was performed using one-way ANOVA. * *p* < 0.05 C vs. H; ** *p* < 0.01 H vs. H + CS; ^&^ *p* < 0.05 H vs. H + CZ and ^#^ *p* < 0.05 H vs. H + CS + CZ.

## Data Availability

The authors confirm that the data supporting the findings of this study are available within the article. The datasets of this study are available from corresponding author upon reasonable request.

## Supplementary Materials

The following supporting information can be downloaded at: https://www.mdpi.com/article/10.3390/molecules31132212/s1.

## Abbreviations

Systemic arterial hypertension (SAH), reactive oxygen species (ROS), including superoxide anion (O2^●−^), peroxynitrite (ONOO-), transient receptor potential vanilloid type 1 (TRPV1), Capsaicin (CS), capsazepine (CZ), and myocardial injury.
