# Supplementary figures and images for: TRPV1 Activation Is Associated with Improved Mitochondrial Function and Cardioprotection in Experimental Hypertension
Source: Molecules. 2026 Jun 23;31(13):2212. doi: 10.3390/molecules31132212 (PMC13363412; doi:10.3390/molecules31132212)

## BAX

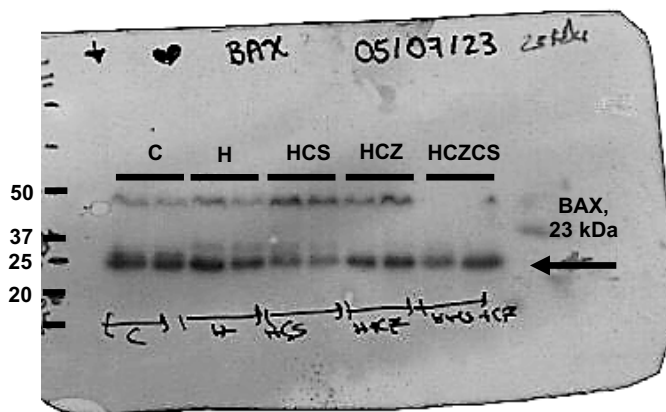

## COX IV

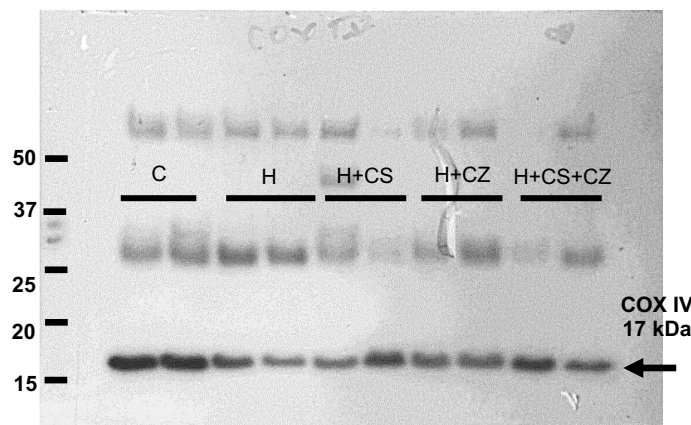

## BAX

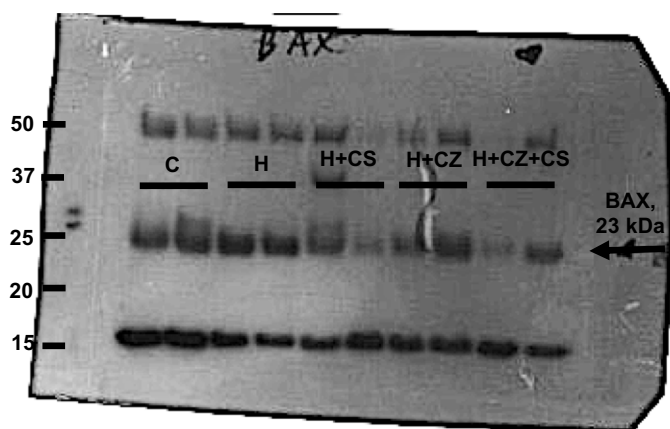

## COX IV

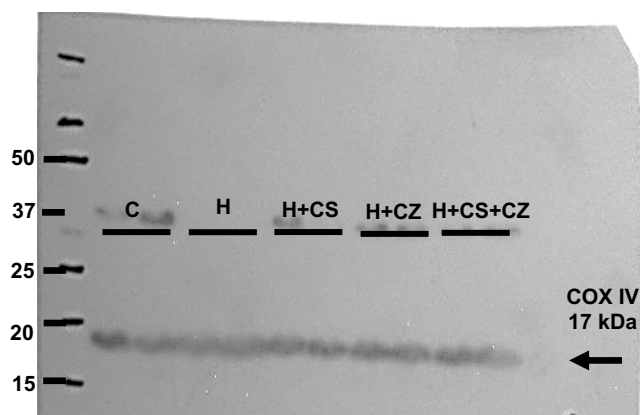

Apaf 1

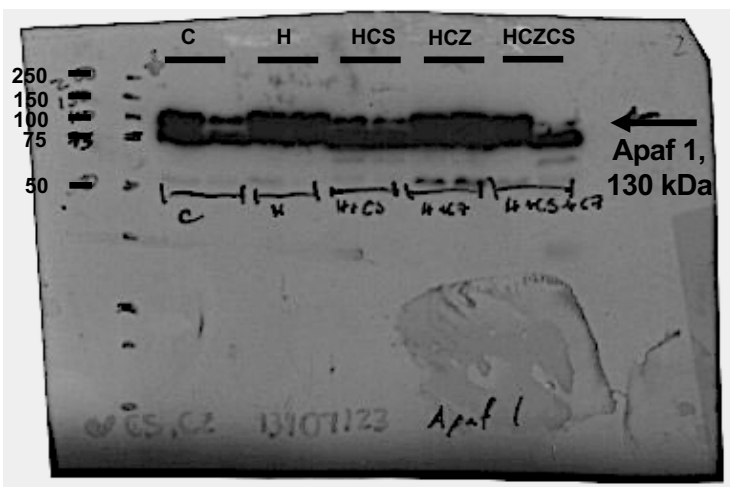

ANT

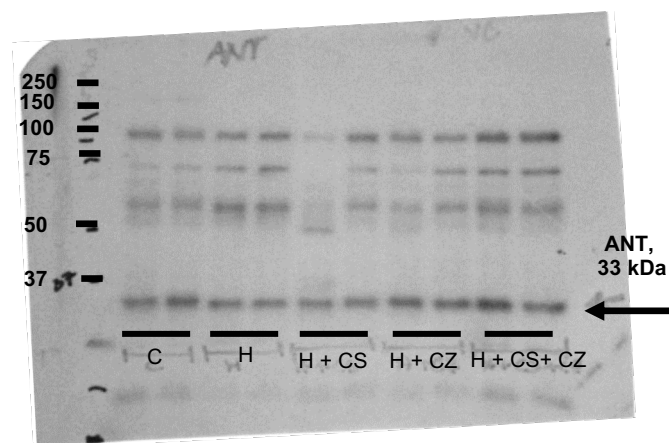

Apaf 1

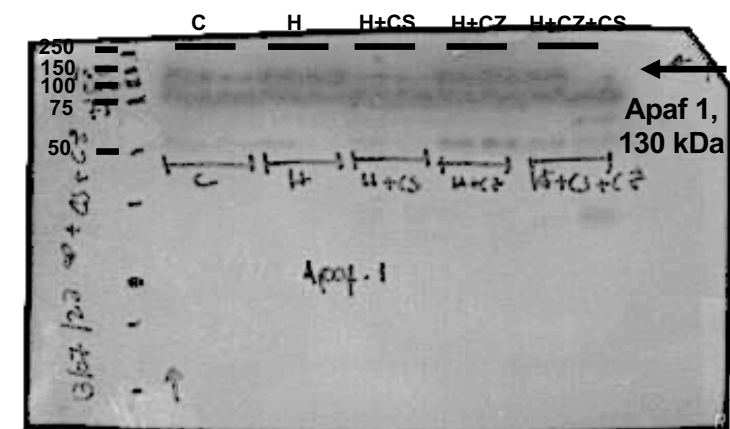

ANT

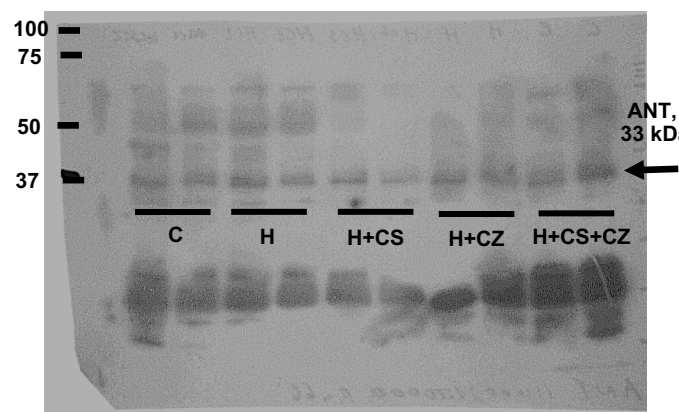

Cit c

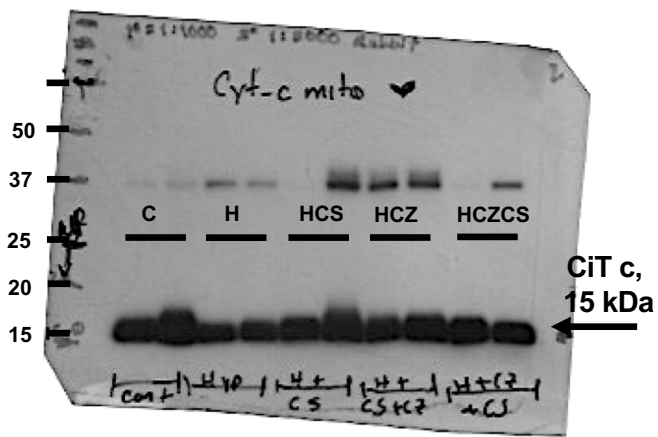

ANT

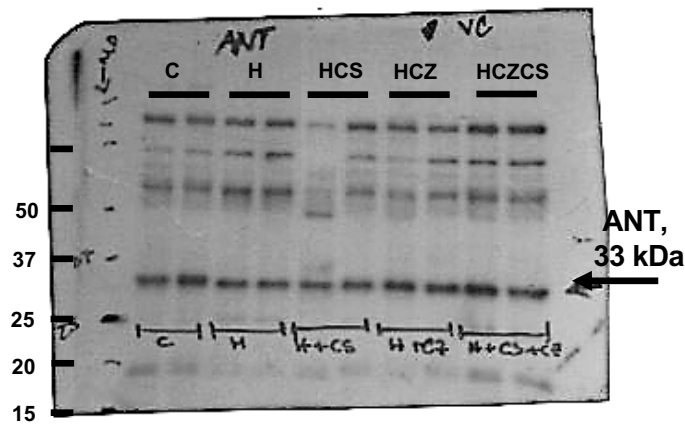

Cit c

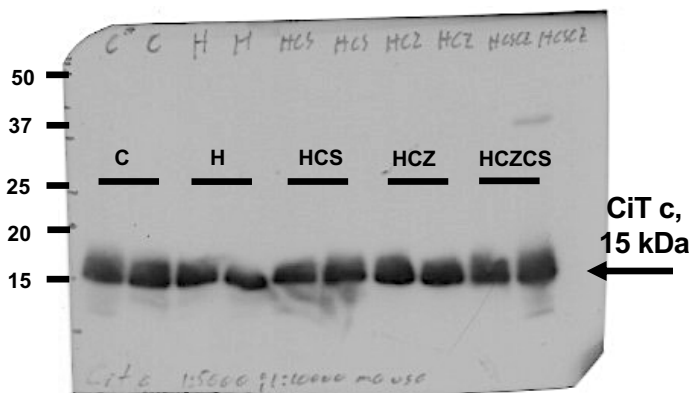

ANT

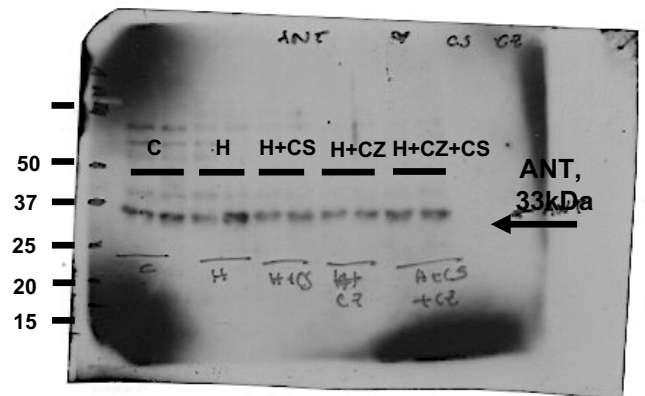

AIF

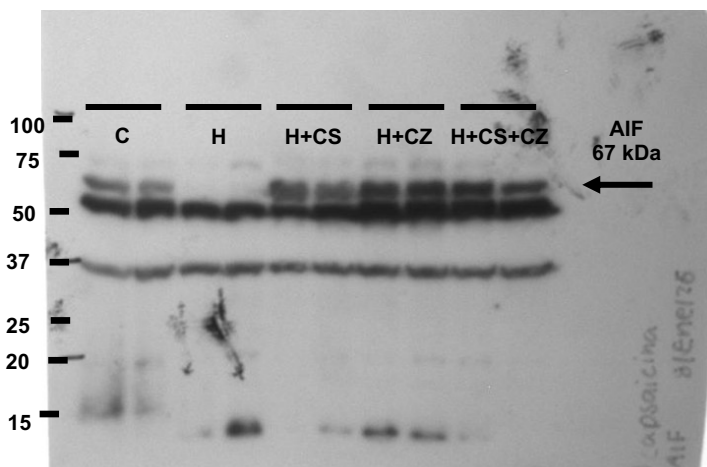

ANT

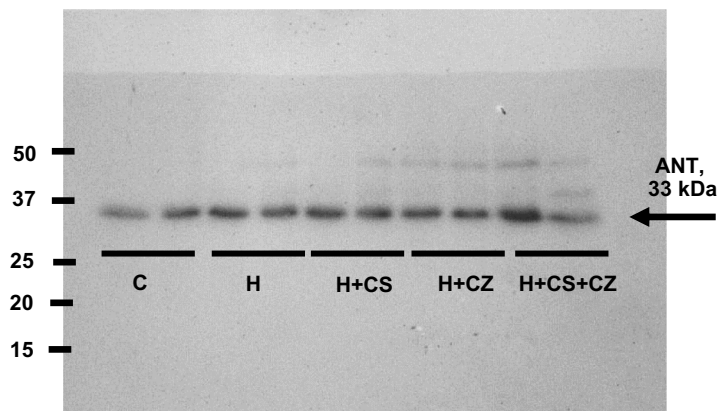

AIF

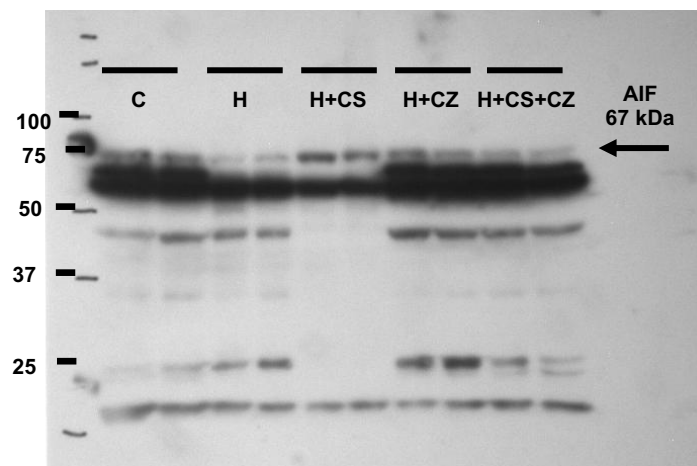

ANT

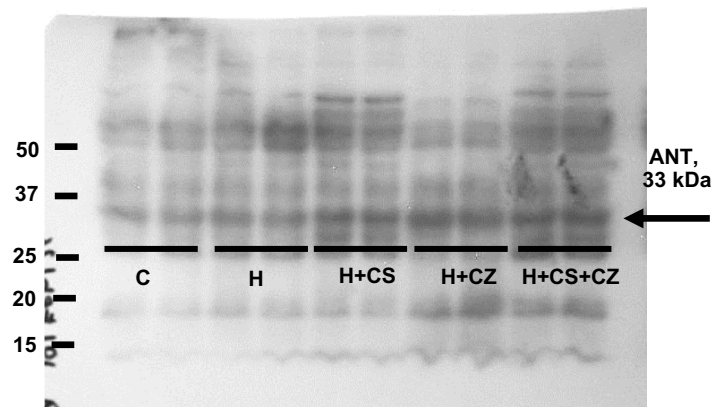

n = 4

Supplement: Supplementary file 1 [file molecules-31-02212-s001.zip › molecules-4328636-supplementary.pdf]
